# Transcriptome Time-Course Analysis in the Whole Period of Cotton Fiber Development

**DOI:** 10.3389/fpls.2022.864529

**Published:** 2022-04-05

**Authors:** Juncheng Zhang, Huan Mei, Hejun Lu, Rui Chen, Yan Hu, Tianzhen Zhang

**Affiliations:** Zhejiang Provincial Key Laboratory of Crop Genetic Resources, College of Agriculture and Biotechnology, Institute of Crop Science, Zhejiang University, Plant Precision Breeding Academy, Hangzhou, China

**Keywords:** *Gossypium hirsutum*, *Gossypium barbadense*, RNA-seq, fiber development, time-course

## Abstract

*Gossypium hirsutum* and *Gossypium barbadense* are the widely cultivated tetraploid cottons around the world, which evolved great differences in the fiber yield and quality due to the independent domestication process. To reveal the genetic basis of the difference, we integrated 90 samples from ten time points during the fiber developmental period for investigating the dynamics of gene expression changes associated with fiber in *G. hirsutum* acc. TM-1 and *G. barbadense* cv. Hai7124 and acc. 3-79. Globally, 44,484 genes expressed in all three cultivars account for 61.14% of the total genes. About 61.39% (*N* = 3,412) of the cotton transcription factors were involved in fiber development, which consisted of 58 cotton TF families. The differential analysis of intra- and interspecies showed that 3 DPA had more expression changes. To discover the genes with temporally changed expression profiles during the whole fiber development, 1,850 genes predominantly expressed in *G. hirsutum* and 1,050 in *G. barbadense* were identified, respectively. Based on the weighted gene co-expression network and time-course analysis, several candidate genes, mainly involved in the secondary cell wall synthesis and phytohormones, were identified in this study, underlying possibly the transcriptional regulation and molecular mechanisms of the fiber quality differences between *G. barbadense* and *G. hirsu*tum. The quantitative real-time PCR validation of the candidate genes was consistent with the RNA-seq data. Our study provides a strong rationale for the analysis of gene function and breeding of high-quality cotton.

## Introduction

*Gossypium* spp. is one of the most important economic crops, accounting for one-third of the total annual fiber demands in the world (Huang et al., 2021). Cotton with cellulose-enriched mature fibers is the largest source of natural textiles. Recently, the two most widely cultivated cotton species have been successfully sequenced and assembled, including *Gossypium hirsutum* (Li et al., 2015; Zhang et al., 2015; Hu et al., 2019; Wang et al., 2019c; Yang et al., 2019; Chen et al., 2020; Huang et al., 2020) and *Gossypium barbadense* (Yuan et al., 2015; Hu et al., 2019; Wang et al., 2019c; Chen et al., 2020) genomes. *G. hirsutum* produces high yields with moderate fiber quality, constituting more than 95% of the world’s cotton production (Li et al., 2015; Hu et al., 2019), whereas *G. barbadense* is renowned for its exceptionally high-quality fibers. The two cotton species have evolved from the same origin with independent domestications (Fang et al., 2017; Huang et al., 2020), resulting in great differences in the plant morphology, yield, fiber quality, and environmental adaptability.

Cotton fibers are extremely elongated single cells derived from the epidermal cells of the ovule. Fibers, which are economically important develop in the ovule epidermal cells –2 to 0 days post-anthesis (DPA), and ultimately reach 2.5–3.5 cm in length. Cotton fiber development is a complicated process consisting of five consecutives but overlapping stages containing cell initiation and differentiation (–3 to 3 DPA); rapid elongation (3–16 DPA); transitional wall thickening; primary cell wall (PCW) remodeling (16–20 DPA); secondary cell wall (SCW) biosynthesis (20–40 DPA); and maturation (40–50 DPA) (Haigler et al., 2012). Compared to *G. hirsutum* (elongation from 5 to 25 DPA), longer fibers were developed, most likely due to the extended elongation phase (5–30 DPA) in *G. barbadense* (Hu et al., 2019). Multiple omics research on cotton fiber have been undertaken on *G. hirsutum* and *G. barbadense*, and it is an important aim for cotton breeding to understand the genetic basis of the difference between the two cotton species for exploring excellent genes controlling key traits during fiber development.

Focusing on two key developmental time points in cotton, the peak of fiber elongation (10 DPA) and the transition to SCW synthesis (22 DPA), the expressed sequence tag (EST) data indicated the differentially expressed genes (DEGs) at 22 DPA included tubulins, cellulose, and sucrose synthases showed a higher expression in *G. barbadense* than *G. hirsutum* (Lacape et al., 2012). As previously mentioned, the gene expression variation between *G. hirsutum* and *G. barbadense* decreased from 7 to 21 DPA, and 20–30% more genes were more highly expressed during fiber elongation (11 DPA) than during SCW thickening (21 DPA) in their microarray analysis (Al-Ghazi et al., 2009). Compared to the transcriptomes and metabolomics of *G. barbadense* and *G. hirsutum* at 10, 15, 18, 21, and 28 DPA, it reveals that a discrete developmental stage of transitional cell wall remodeling occurs before the secondary wall cellulose synthesis begins (Tuttle et al., 2015). Chen et al. (2012) analyzed the microarray data of TM-1 and Hai7124 fiber at five developmental time points (5, 10, 15, 20, and 25 DPA), and considered the high-level and long-term expression of positive regulators, including auxin and cell wall enzyme genes, for fiber cell elongation at 10 and 25 DPA may result in the determination of *G. barbadense* fiber continuing elongation.

Nowadays, the accumulating evidence supports that the regulation of RNA transcription significantly plays pivotal functions in plant growth and development. To date, RNA-seq has been widely employed in the crops to understand its response to drought, salinity, developmental stages, verticillium wilt, useful to analyze the gene expression and molecular mechanisms underlying various traits (Shankar et al., 2016; Li et al., 2017a; Aleem et al., 2021). Based on the analysis of the transcriptome profiling between drought-tolerant and sensitive soybean genotype, 10 DEGs were recognized as putative candidate genes modifying drought tolerance in soybean (Aleem et al., 2021). In rice, transcriptomes comparison of a drought-tolerant (N22) and a salinity-tolerant (Pokkali) rice cultivar revealed some common and exclusive metabolic pathways, which may be important for desiccation and/or salinity stress tolerance (Shankar et al., 2016). Through multiple comparisons of 15 samples between two chromosome segment substitution lines at 10 and 28 DPA, two peroxidases and four flavonoid pathway-related genes were identified, which could play a role in fiber development and quality formation (Li et al., 2017a). Due to transcription which is a highly dynamic biological process, it is vital to study gene regulation associated with a particular biological process at more than one time point. In the previous study of rice, time-course RNA-seq analysis across the three time points (1, 7, and 14 DPA) provides an improved understanding of gene regulation during the formation of nodule-like structures (Thomas et al., 2020). A high temporal-resolution transcriptome landscape was constructed using data for 31 time points to unravel the genetic control of maize seed development, with 31,256 interactions among 1,317 transcription factors (TFs) and 14,540 genes (Yi et al., 2019). For study on fiber development in cotton, analyzing and mining expression data at more time points are of great importance. However, relatively few transcriptome studies have investigated at multiple time points underlying the differences of phenotype during the fiber development between the two major cultivated species *G. hirsutum* and *G. barbadense*.

In this study, RNA-seq analysis of high-density time points during the fiber developmental period was performed in three cotton cultivars, *G. hirsutum* acc. TM-1 and *G. barbadense* cv. Hai7124 and acc. 3-79. A comprehensive method with time-course analysis and weighted gene co-expression network (WGCNA) was employed in the global analysis. We focus on the differential gene expression profiles during cotton fiber development, which may underly possibly the transcriptional regulation and molecular mechanisms of the fiber quality differences between *G. barbadense* and *G. hirsutum*.

## Materials and Methods

### Plant Materials

Three cotton cultivars, in *G. hirsutum* acc. TM-1 and *G. barbadense* cv. Hai7124 and acc. 3-79, were planted in the Maanshang Breeding Station of Zhejiang University (MBS/ZU), Anhui, China, in 2020. Around the anthesis, buds or balls were collected in –3, –1, 0, 1, 3, 5, 10, 15, 20, and 25 DPA, the vital developmental stages of fiber from three cultivars. A total of 90 samples, including three biological replicates, were collected for each developmental time point in the three cultivars. The ovule or fiber (–3 to 5 DPA for ovule, 10–25 DPA for fiber) was immediately stripped out after sampling and then was dropped to liquid nitrogen for fast freezing. The ovule or fiber samples were t temporarily stored in –80°C prepared for RNA extraction.

### RNA Extraction and Transcriptome Sequencing

According to the EASYspin Plus plant RNA rapid extraction kit (#RK16, Molfarming), we extracted RNA of the 90 samples from three cotton cultivars. The RNA samples were built into libraries for sequencing according to the Illumina transcription library manual. High-throughput sequencing was performed with a 150-bp paired-end strategy on the Illumina Novaseq platform of Beijing BerryGenomics Co., Ltd. A total of 365 Gb of transcriptomic sequence data were generated finally with an average of 4.05 Gb for each library.

### Processing of Data and Statistical Analysis

For raw data filtering, Fastp (version 0.19.4) (Chen et al., 2018) was performed to control quality and output the clean reads. Then, the paired-end clean reads were aligned against the reference genome sequence (*G. hirsutum* acc. TM-1, version 2.1) (Hu et al., 2019) with the Hisat2 (version 2.2.1) (Kim et al., 2015). The gene expression abundance of the samples was calculated using Stringtie (version 2.1.4) (Pertea et al., 2015) and the fragments per kilobase of exon per million fragments mapped (FPKM) value for each gene were obtained, which was used as the measure of the gene expression level. The mapped ratio of a replicate sample of Hai7124 at 25 DPA was only 55.66%, so it was eliminated in the subsequent analysis (Supplementary Table 1). In this study, the genes with FPKM >1 were considered as the expressed genes. The principal component analysis (PCA) and Pearson’s correlation coefficient (PCC) analysis were performed using Prcomp and Cor functions in R (version 3.6.4). We conducted the Gene Ontology (GO) analysis by the R package ClusterProfiler (version 3.14.3) (Yu et al., 2012). The GO terms exhibiting a *p*-value of < 0.05 were significantly enriched. The corresponding phenotype data were respectively retrieved from http://mascotton.njau.edu.cn/info/1058/1132.htm (Fang et al., 2017), and http://cotton.zju.edu.cn/download.html (Fang et al., 2021). The identification of the TF families in *G. hirsutum* gene members was performed by Hu et al. (2019).

### Analysis of Differentially Expressed Genes

Taking *G. hirsutum* acc. TM-1 (version 2.1) as the reference genome (72,761 genes), the transcription genes were annotated for three cotton cultivars, *G. hirsutum* (TM-1) and *G. barbadense* (Hai7124, 3-79). The differentially expressed genes (DEGs) were identified using DESeq2 (version 1.26.0) (Love et al., 2014) with the threshold of 2-fold expression changes [| log_2_(fold-change) | ≥ 1] and false discovery rate (FDR) < 0.05.

### Time-Course Analysis

MaSigPro (version 1.58.0) (Conesa et al., 2006) was used for the time-course analysis. Setting *G. hirsutum* (TM-1) as the control check, we input the expressed gene matrix of all the samples. Then, the design matrix was constructed according to the three cotton cultivars and ten developmental time points. The degree of polynomial regression in this study was set to 9, and the forward elimination algorithm was used to perform stepwise regression, with R-squared >0.7 as the threshold to extract the significant fitting genes as the time-course DEGs. Hierarchical clustering (hclust) was performed based on the correlation coefficient distance.

### Weighted Gene Co-expression Network Construction

Co-expression networks were constructed using the WGCNA (version 1.70) (Langfelder and Horvath, 2008). Time-course DEGs of *G. barbadense* (*N* = 1,050) and *G. hirsutum* (*N* = 1,850) were used for the WGCNA unsigned co-expression network analysis, respectively. The modules were obtained using the automatic network construction function *blockwiseModules* with default settings, except that the power is 6 (*G. hirsutum*) and 13 (*G. barbadense*), minModuleSize is 30, and mergeCutHeight is 0.25. Among the identified modules, the gray module representing the failed classified genes was considered as an invalid module. The top five genes of kME (Module eigengene-based connectivity) were selected as the hub genes. The interaction network among the hub genes and their associated genes (top 50 of kME, and weight value > 0.10) was visualized using Cytoscape (version 3.9.0) (Otasek et al., 2019).

### Merging Results From MaSigPro and Weighted Gene Co-expression Network

The genes clustering by MaSigPro were based on their similar expression profiles, while the algorithm of WGCNA was based on the correlation among the genes. To get the common genes selected by these two approaches, we integrated the clustering results of time-course with the co-expression network modules. Then, new modules were constructed with an overlapping rate >70%.

### Quantitative Real-Time PCR Verification of Candidate Genes

Quantitative real-time PCR (qRT-PCR) was employed to verify the expression pattern of the candidate genes from the transcriptome data. We used HiScript^®^ II Q RT SuperMix for qPCR (+gDNA wiper) kit (Vazyme, R223-01) to reverse transcribe 1 μg RNA of each sample into cDNA. qRT-PCR was conducted in StepOnePlus (Applied Biosystems), with three replicates for each sample. All specific primers (Supplementary Table 2) were designed by Primer Premier 5. Amplification procedure: pre-denaturation at 95°C for 5 min; denaturation at 95°C for 10 s, annealing at 60°C for 30 s, repeated for a total of 40 cycles. 2^–ΔΔ*Ct*^ (Livak and Schmittgen, 2001) was used for calculating the relative expression level of the gene.

## Results

### Transcriptome Sequencing of Whole Fiber Development

Given that the phenotypes data of three cotton cultivars in the previous study, we have knowledge that *G. barbadense* cv. Hai7124 and acc. 3-79 have excellent fiber quality, and the latter is of better quality, while *G. hirsutum* acc. TM-1 with high yield traits (Fang et al., 2017, 2021; Supplementary Table 3). To reveal the relation of the gene expression profile with the difference in the fiber between *G. hirsutum* and *G. barbadense*, 90 RNA-seq libraries of ovule or fiber samples were constructed and sequenced at –3, –1, 0, 1, 3, 5, 10, 15, 20, and 25 DPA in three cotton cultivars. After filtering the low-quality reads, a total of 365 Gb clean data were obtained, of which the average clean reads per sample were 25.42 million, together with not less than 88.68% of the Q30 and over 42.97% of the GC content (Supplementary Table 1). Through alignment analysis, 98.17–99.56% of the clean data was mapped on the genome of *G. hirsutum*, which implied a reliable quality of the RNA-seq (Supplementary Table 1).

The PCA showed that the biological replicates of three cotton cultivars have good consistency, and the same developmental stage of the three materials clustered together, indicating the difference between developmental stages was greater than that between the cultivars (Supplementary Figure 1). A number of three main clusters were divided among all samples, which exactly corresponded with the fiber developmental process that the initiation stage (–3 to 1 DPA), rapid elongation stage (3–10 DPA), primary wall remodeling, and secondary wall thickening stage (15–25 DPA) (Supplementary Figures 2A–C). This is basically consistent with a previous study on cotton fiber development (Hu et al., 2019). PCC analysis was adopted to uncover the correlations between *G. hirsutum* and *G. barbadense*, which produced one heatmap showing the two species have basically common fiber developmental stages approximately, including into three parts, consistent with the PCA (Supplementary Figure 2). Though the transcriptome data of *G. hirsutum* and *G. barbadense* showed the most consistency in the developmental process, there was also a gene expression bias (Supplementary Figures 2D,E). For example, the gene expression pattern of TM-1 at 20 DPA has a higher correlation with the 25 DPA of 3-79 (Pearson’s *r* = 0.85) and Hai7124 (Pearson’s *r* = 0.84) than the TM-1 at 25 DPA. This result means that *G. barbadense* has an extensional period for fiber growth and potentially leads to longer fiber than *G. hirsutum* (Hu et al., 2019; Supplementary Figures 2D,E).

### Expressed Genes Play a Critical Role in Complex Fiber Developmental Process

**FIGURE 1:**
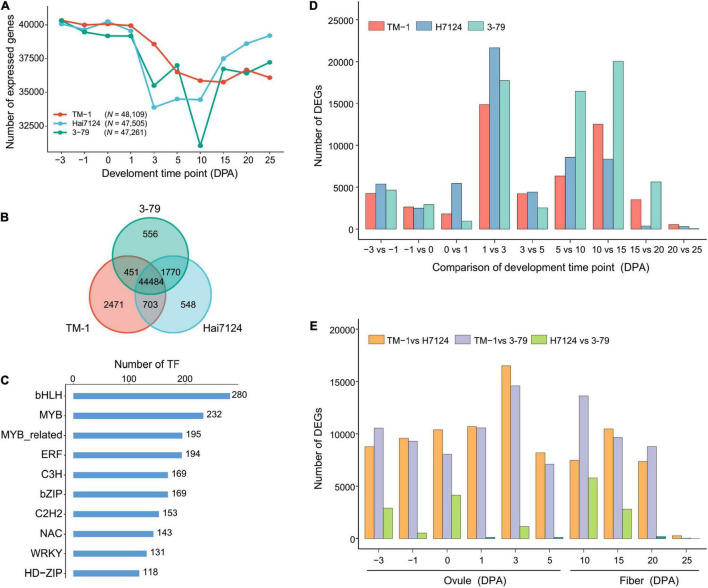
Statistical analysis of expressed genes, TFs, and DEGs during fiber development. **(A)** Line charts showing the number of expressed genes during different fiber developmental stages in TM-1, 3-79, Hai7124. *N* represents the total number of expressed genes for the cultivar. **(B)** Venn diagram of the expressed genes. Each color represents one cultivar. **(C)** Frequency of the transcription factors among 44,484 expressed genes. Each number represents the number of gene families. **(D)** Statistics of DEGs within three cotton cultivars. **(E)** Statistics of DEGs between three cotton cultivars.

Cotton fiber development is a dynamic and sophisticated developmental process orchestrated by complicated regulatory networks involving a large number of genes. Throughout the period of fiber development, *G. barbadense* (Hai7124, 3-79) started an increase in the number of expressed genes from 15 DPA (Figure 1A). The later period of fiber development (15–25 DPA) is the main stage for the fiber quality formation, when more genes become active and should be associated with the extra-long fibers in *G. barbadense* (Hu et al., 2019). In total, 48,109 and 47,505 and 47,261 expressed genes were identified in TM-1, Hai7124, and 3-79, respectively, with 44,484 genes expressed in all three cultivars accounting for 61.14% of the total genes (Figure 1B). Due to the enormous diversity and functional complexity, TFs as an important class of genes are involved in processes, such as metabolic regulation, plant growth and development, and response to environmental signals (Wang et al., 2016; Hoang et al., 2017; Romani and Moreno, 2021). Moreover, 3,412 out of the 44,484 expressed genes were identified as TFs, which consist of 58 TF family, including all the known TF families in cotton (Supplementary Table 4). In detail, we found 61% of the total bHLHs (280 out of 459) plays an extensive role in cotton development (Liu et al., 2018), 52.61% MYBs (232 out of 441) related to the regulation of SCW thickening (Dubos et al., 2010; Salih et al., 2016; Huang et al., 2019), and 43.79% ERFs (194 out of 443) involved in hormone biosynthesis and signal transduction (Vissenberg et al., 2005; Feng et al., 2020; Figure 1C and Supplementary Table 4).

Under the thresholds of absolute log_2_(fold-change) ≥ 1 and FDR < 0.05, DEGs between two adjacent developmental periods showed a dramatical increase during the 1–3 DPA stage (TM-1, *N* = 14,859; Hai7124, *N* = 21,641; 3-79, *N* = 17,722) (Figure 1D and Supplementary Table 5), implicating the dramatic gene expression pattern changes which should be required for driving fiber morphogenesis at the fast-elongation stage. Interestingly, the difference between *G. hirsutum* and *G. barbadense* (TM-1 vs. Hai7124, *N* = 16,497; TM-1 vs. 3-79, *N* = 14,595) at 3 DPA was obvious (Figure 1E and Supplementary Table 6), indicating that the fast-elongation stage of fiber development plays a remarkable role to the differences of fiber quality between *G. hirsutum* and *G. barbadense.* Combined with the previous results of PCA and PCC analysis (Supplementary Figure 2), 3 DPA was located near the critical point of initiation stage (–3 to 1 DPA) and rapid elongation stage (3–10 DPA), suggesting that the genes expressed in transitional stages involved complex regulations during fiber development.

### Time-Course Differential Expression Analysis Between *Gossypium hirsutum* and *Gossypium barbadense*

**FIGURE 2:**
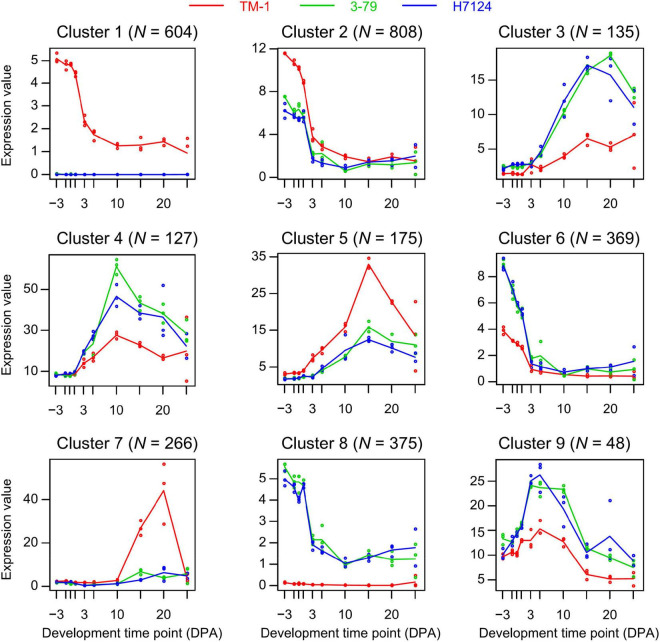
Different gene expression patterns based on the time-course analysis. Each cluster represents a trend of gene expression and the number on top indicates the number of genes in cluster. Different colored curves represent cultivars and each curve represents the median profile of genes at different developmental time points.

To investigate the diverse expression pattern during ten time points in three cotton cultivars, we applied the MaSigPro analysis to our data. Here, we identified 4,545 and 4,648 time-course DEGs (significance with FDR < 0.05 and R-squared threshold > 0.7 for multiple tests) in two comparisons of *G. hirsutum* and *G. barbadense* (TM-1 vs. Hai7124, TM-1 vs. 3-79), respectively. The cluster of genes that shared a similar expression pattern in the two comparisons was considered as a representative pattern of land-sea cotton differences. Therefore, the intersection genes (*N* = 2,900) of each similar expression pattern were used for follow-up analysis. A total of nine clusters corresponding to the differential expression patterns were identified by the hclust method (Figure 2 and Supplementary Table 7).

**FIGURE 3:**
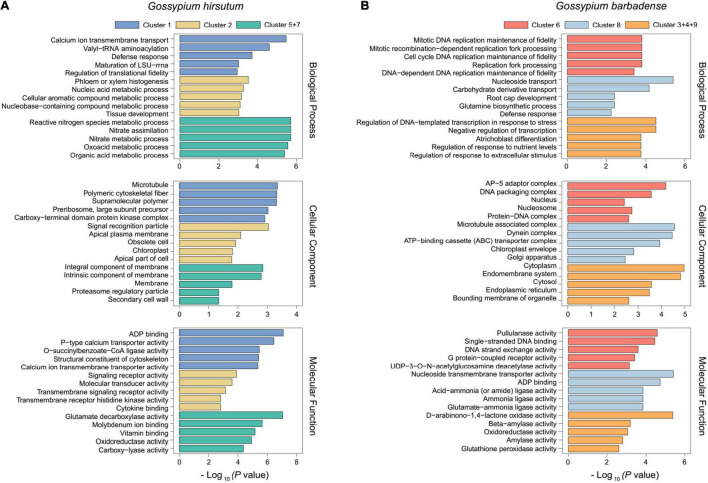
GO enrichment analysis results of predominant expression in *G. hirsutum* and *G. barbadense*. GO enrichment analysis results of predominant expression in *G. hirsutum* **(A)** and *G. barbadense* **(B)**, including the biological process, cellular component, and molecular function. The different colors represent different clusters. The X–axis represents -log_10_(*p*–value) and the enriched GO terms are indicated on the Y-axis.

After the time-course analysis clustering, the dynamic expression pattern was divided into two dominant expression types as a whole. The first one was the dominant expression pattern of *G. hirsutum* (*N* = 1,850), represented by Cluster 1, Cluster 2, Cluster 5, and Cluster 7. The second one was *G. barbadense* (*N* = 1,050) represented by Cluster 3, Cluster 4, Cluster 6, Cluster 8, and Cluster 9 (Figure 2). To further uncover the potential functions, GO enrichment for each cluster was performed. Among them, we observed that most of the genes were enriched in cytoskeleton structure, ADP binding, calcium ion transport, and signal transduction terms that were closely related to plant cell morphogenesis (Lloyd, 1989; Qian and Xiang, 2019) in Cluster 1 (*N* = 604), which represents a group of genes that were nearly devoid of expression in *G. barbadense* (Figure 3A). In contrast, the genes in Cluster 8 were predominantly expressed in *G. barbadense*, of which the terms involved in nucleoside transmembrane transporter activity, amide ligase activity (Figure 3B). In Cluster 5 (*N* = 807), *G. hirsutum* was more actively expressed at the initial stage of fiber development (–3 to 1 DPA) than *G. barbadense*, and we observed several GO terms were enriched in transmembrane-signaling receptor activity, nucleic acid metabolism, tissue development, which may relate to the ovule epidermal cells division and differentiation (Hou et al., 2020) in the early fiber development (Figure 3A). Additionally, we found that the genes of *G. barbadense* in Cluster 6 have the upper hand when they expressed during the initial stage of fiber development, most of which were involved in biological process and molecular functions, including DNA replication, mitosis, nucleosomes, and G protein-coupled receptor activity, which associate with cell proliferation (Figure 3B). Notably, we observed that Clusters 5 and 7 (*N* = 449) represented the specific expression patterns of *G. hirsutum* at 15 and 20 DPA, respectively, the dominant subcategories in cellular component and molecular function were membrane and SCW (Lamport et al., 2018), and correlated with the metabolism of active nitrogen, nitrate, and organic acid (Figure 3A). Furthermore, the gene expression of Clusters 3, 4, and 9 (*N* = 307) prevailed in *G. barbadense* at 15, 10, and 5 DPA, respectively, the GO terms included mainly the negative regulation of transcription under stress, response to extracellular stimulus, and the regulation of nutrient levels (Figure 3B).

### Co-expression Network Construction and Overlap Analysis With MaSigPro

**FIGURE 4:**
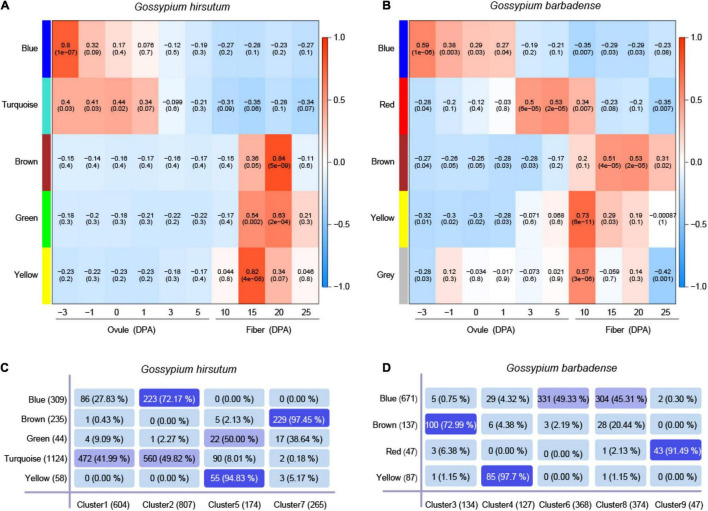
Co-expression network construction and overlap analysis with MaSigPro. **(A,B)** Weighted gene co-expression network analysis (WGCNA) of the genes with dominant expression in *G. hirsutum* (*N* = 1,850) and in *G. barbadense* (*N* = 1,050) at ten time points of fiber development, respectively. Each row represents a module, and the correlation coefficient and the *p*-value calculated by Fisher’s exact test are shown in each square. The table is color-coded by correlation according to the color legend. The intensity and direction of correlations are indicated on the right of the heat map (red, positive; green, and negative). **(C,D)** Overlapping analysis of genes in the four clusters and five modules in *G. hirsutum*, while genes in the five clusters and four modules in *G. barbadense*.

Based on genes predominantly expressed in *G. hirsutum* (*N* = 1,850) and *G. barbadense* (*N* = 1,050), the co-expression networks were constructed by WGCNA to find a correlation between the modules and gene expression (Supplementary Tables 8, 9). In the co-expression network of *G. hirsutum*, five modules were identified, harbored 127 TFs, consisting of 36 (blue module), 63 (turquoise module), 20 (brown module), 6 (green module), and 1 (yellow module) (Supplementary Table 8). Removing gray module considered as the invalid module in *G. barbadense*, four modules were identified, harbored 50 TFs consisting of 38 (blue module), 8 (brown module), 2 (red module), and 2 (yellow module) (Supplementary Table 9). The TFs represented >10% of the total genes included in some of the modules, indicating tight regulation of the transcriptional activity. Further, in the co-expression network of *G. hirsutum*, the blue module was significantly correlated with –3 DPA (Pearson’s *r* = 0.80), brown module correlated with 20 DPA (Pearson’s *r* = 0.84), and yellow module correlated with 15 DPA (Pearson’s *r* = 0.82) (Figure 4A). In the co-expression network of *G. barbadense*, the yellow module was significantly correlated with 10 DPA (Pearson’s *r* = 0.73), the red module specifically correlated with 3–10 DPA (Figure 4B).

The overlapping analysis of the genes in the four clusters from MaSigPro and five modules from WGCNA was performed in *G. hirsutum*, whereas overlapping of genes in five clusters and four modules was performed in *G. barbadense* (Figures 4C,D). The results showed that each module overlapped with a specific cluster. For example, the overlapping rate of genes between the brown module and Cluster 7 reached 97.45% in *G. hirsutum* (Figure 4C). The overlapping rate of the genes between the yellow module and Cluster 7 reached 97.70% in *G. barbadense* (Figure 4D). The WGCNA and MaSigPro results were in agreement (Figures 4C,D). The genes that appeared simultaneously in modules and clusters were considered important in the biological function of fiber development. Then, these genes were regrouped into new modules, with the threshold of an overlapping rate >70%. As a result, three new modules, Gh-Cluster2-Blue, Gh-Cluster5-Yellow, and Gh-Cluster7-Brown, were generated in *G. hirsutum* (Supplementary Table 10), and three new modules were generated in *G. barbadense*, including Gb-Cluster3-Brown, Gb-Cluster4-Yellow, and Gb-Cluster9-Red (Supplementary Table 11).

The GO enrichment analysis observation is possibly indicative of certain functions or pathways that were prominently aggregated in the new modules, such as organic acid metabolic process (GO:0006082, *p* = 7.2 × 10^–9^) and molybdenum ion binding (GO:0030151, *p* = 1.64 × 10^–7^) in Gh-Cluster7-Brown (Supplementary Figure 3B), ubiquitin-protein transferase activity (GO:0004842, *p* = 7.18 × 10^–4^), and cell wall biogenesis (GO:0042546, *p* = 1.29 × 10^–3^) in the Gb-Cluster3-Brown module (Supplementary Figure 3C), indicating that the genes of the new modules showed more representative functions in the biological process.

### Identification and Validation of Candidate Genes

Based on the value of eigengene connectivity, five top-ranked genes with the highest value of kME in each of the six new modules were selected as the hub genes. The hub genes did not fully represent the biological function of each module, so we also needed to pay attention to the functions of the other genes in the co-expression network. To intuitively observe the genes in each module, the interaction networks of the high-weight value pairs were visualized using Cytoscape (Otasek et al., 2019; Supplementary Figure 4).

Highly related to –3 DPA, Gh-Cluster2-Blue (*N* = 223) module had two disease-resistant protein genes (*GH_A10G2546*, *GH_D05G3659*), which implied to the wide adaptability of *G. hirsutum* at the early stage of fiber development. It is worth noting here that we found a gene *GhSusA1* (*GH_D08G1434*) in Gh-Cluster7-Brown related to 20 DPA had been reported, which is previously linked to fiber development, whose overexpression promoted the thickening and strengthening in the secondary wall of the fiber cells (Jiang et al., 2012; Li et al., 2016). To further investigate the function of correlative genes in three *G. hirsutum* networks, we observed two auxin response factors (ARFs) genes (*GH_A06G0864*, *GH_A08G2737*), which served as critical components of auxin signaling and mediated auxin regulation of diverse physiological processes in the cotton fiber development. *GH_D09G1632* was annotated as the glycosyltransferases, which involves in the synthesis of glucuronoxylan hemicellulose in SCW, which may participate in cell wall morphogenesis (Supplementary Figures 4A–C). Apart from these, 33 and 20 TFs like bZIP, HD-ZIP, and MYB were identified in Gh-Cluster2-Blue and Gh-Cluster7-Brown, respectively (Supplementary Table 10).

Gb-Cluster3-Brown (*N* = 100), Gb-Cluster4-Yellow (*N* = 85), Gb-Cluster9-Red (*N* = 43) were the dominant expression submodule of *G. barbadense*, which correlated with 20, 10, and 5 DPA, respectively. Given that the later period of fiber development was a critical stage that formed differences between *G. barbadense* and *G. hirsutum*, we would focus on the Gb-Cluster3-Brown module correlated with 20 DPA. In this module, we identified that several hub genes, such as *GH_A12G0677*, were annotated as LIM zinc finger domain-containing protein, and that Dof TF *GH_D10G0298* with zinc finger domain may involve in the regulation of cellulose biosynthesis (Supplementary Table 12). Coincidentally, the gene *GH_D05G2692* encoding cellulose synthase was also identified in the Gb-Cluster3-Brown module. It suggested that they likely jointly participate in the formation of fiber fineness and strength quality of *G. barbadense*. Noteworthy, we found a gene *GH_D06G0983* in Gb-Cluster3-Brown involving in Xyloglucan endotransglucosylase/hydrolase (XTH), which could promote wall loosening to strengthen cell walls (Michailidis et al., 2009). In addition, we also found three genes (*GH_D13G0580*, *GH_A03G1264*, *GH_D09G1018*) were annotated as calcium-dependent protein kinase 26-like, biosynthesis and degradation of lignin and auxin, Cu-Zn superoxide dismutase in the other two networks of *G. barbadense*, which may be active in fiber elongation. And 11 TFs like WRKY, TCP, and NAC were also identified in these networks (Supplementary Table 11).

**FIGURE 5:**
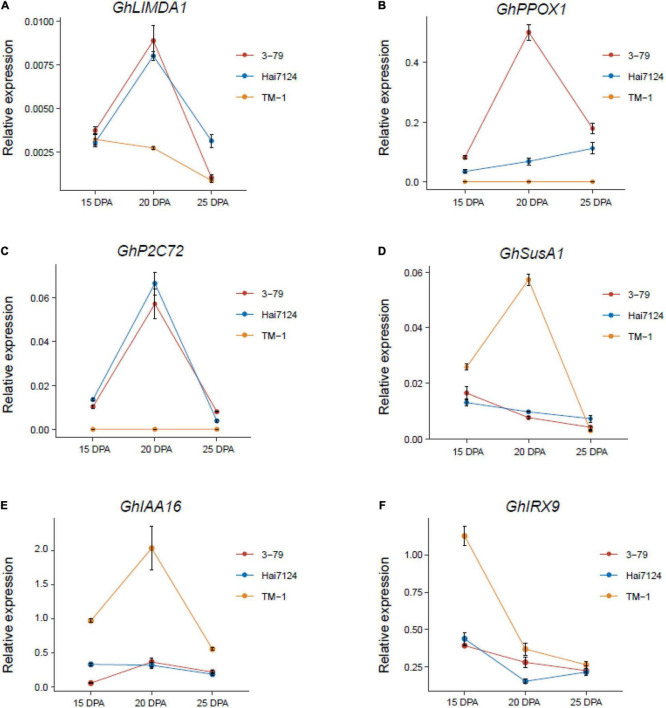
Validation of the candidate genes with qRT-PCR. **(A–F)** The expression levels of six candidate genes in 15, 20, and 25 DPA fibers of TM-1, Hai7124, and 3-79 were performed with qRT-PCR. Each point of the line chart shows the average value of three repeats, and each error bar represents one standard error.

To validate the RNA-seq data, six subgenome ortholog pairs were performed by qRT-PCR: *GhIAA16* [*GH_A08G2737, GH_D08G2734*], *GhIRX9* [*GH_A09G1686, GH_D09G1632*], *GhLIMDA1* [*GH_A12G0677, GH_D12G0689*], *GhPPOX1* [*GH_A05G2673, GH_D05G2692*], *GhP2C72* [*GH_A06G0976, GH_D06G0983*], *GhSusA1* [*GH_A08G1412, GH_D08G1434*]. The housekeeping gene *GhY8991* was selected as the reference gene during the validation analysis (Supplementary Table 2). In general, the relative expression patterns of the candidate genes based on qRT-PCR were consistent with the results of RNA-seq, and highly confirmed the credibility and accuracy of the RNA-seq data (Figure 5, Supplementary Figure 5, and Supplementary Table 13).

## Discussion

### Time-Course Experiments Help Us to Characterize Biological Process

With the rapid development of sequencing technology, a large amount of data, diversity of treatment conditions, and the dynamic nature of the experiments pose great challenges to data analysis (Fu et al., 2013; Wang et al., 2019a). Compared to the single-time series investigating, a time-course experiment design offers a more macroscopic and comprehensive view for understanding the transcriptional regulations and molecular mechanisms of the biological process that are still not well-characterized (Bar-Joseph, 2004; Shah and Corbeil, 2011; Hejblum et al., 2015). The comparative analysis for 12 time-course tools revealed that splineTC and MaSigPro better performance on long-time series (>8 time points) data than standard differential expression analysis (Spies et al., 2019). Thus, a two-regression-step approach was defined by MaSigPro to discover genes with significantly changed expression profiles between the control and experimental groups in our study (Conesa et al., 2006). Here, we totally identified 2,900 time-course DEGs between *G. hirsutum* and *G. barbadense*. Subsequently, to further identify the genes with highly correlated expression, based on these time-course DEGs, we employed the WGCNA analysis to construct the co-expression network for dominance expression profiles of *G. hirsutum* and *G. barbadense*, respectively. The GO enrichment analysis of the new modules in *G. hirsutum* and *G. barbadense* was performed and showed good consistency before and after merging (Figure 3 and Supplementary Figures 3A–E). Moreover, the genes in the new modules showed greater significance for certain biological processes or molecular functions. Therefore, our integrated approach, and combining tools like MaSigPro and WGCNA, can efficiently identify a batch of candidate genes related to cotton fiber through the whole development.

### Preferential Expressed Genes Involved in Cell Wall and Phytohormones

The cotton fiber quality traits mainly include fiber length, fiber strength, fiber fineness (micronaire value), and compared with *G. hirsutum*, the fibers of *G. barbadense* are slenderer and have stronger toughness (Ma et al., 2019), while the composition and structure of the plant cell walls are critical for cell strength and extensibility (Zhang et al., 2021b). The main components of the cotton fiber cell walls are cellulose, hemicellulose, and structural proteins, and the changes of contents and types with the growth and development of plants had a non-negligible impact on the mechanical properties of the cell wall (Sandhu et al., 2009). It is well-known that zinc finger-domain proteins are a class of developmental regulators that play important roles in cytoskeletal regulation, gene transcription, and signaling. The XLIM protein (*GhXLIM6*) was confirmed to *via* suppressing transcription of *CesA* gene (*GhKNL1*) to promote cellulose biosynthesis, thereby fine-tuning the SCW formation in the fiber elongation stage (Li et al., 2018). In our study, a pair of genes (*GhPPOX1*, *GhLIMDA1*) were identified in the dominantly expressed genes of *G. barbadense*, which highly correlated with 20 DPA, and may have mutual regulation in the SCW cellulose metabolism pathway. These results implied that the regulation of the cellulose biological process was likely to play a central role in the excellent fiber length quality formation of *G. barbadense*. Xyloglucan of the hemicellulose component is a structural polysaccharide of the plant cell walls, and the cross-linked structure of xyloglucan and cellulose microfibrils is one of the factors hindering cell elongation (Fry et al., 1992; Vissenberg et al., 2005). XTH could indirectly elongate the cells by deconstructing such cross-links to relax the cell wall. To validate the function of XTH in cotton fiber, Lee et al. (2010) confirmed that the over-expressed *GhXTH1* had produced mature cotton fibers that were between 15 and 20% longer than control. Interestingly, we identified a gene (*GhIRX9*) in the modules of *G. hirsutum*, which was annotated as the synthesis of glucuronoxylan hemicellulose in the SCW, while an XTH gene (*GhP2C72*) was discovered in the dominantly expressed genes of *G. barbadense*. In the previous study, the cotton fiber middle lamella (CFML) of *G. barbadense* fiber contained lower levels of xyloglucan compared to *G. hirsutum* fiber, and the xyloglucan endo-hydrolase activity was also higher in the *G. barbadense* fiber (Avci et al., 2013). Thus, we speculated that different expression patterns of these genes related to the SCW may be one of the factors that caused the difference in the fiber quality of *G. hirsutum* and *G. barbadense*.

Biosynthesis and signal transduction pathways that mediate the effects of phytohormones are quite essential for fiber development in cotton (Kim and Triplett, 2001; Ahmed et al., 2018). In the previous studies, auxin could promote fiber initiation and fiber units when applied to the ovules exogenously (Gialvalis and Seagull, 2001), and *GhARF2b*, a homolog of the *AtARF2*, shows a predominant expression in the cotton fiber tissue, and it negatively influences cotton fiber elongation but promotes fiber initiation (Zhang et al., 2021a). In the Gh-Cluster2-Blue module, which is associated with –3 DPA, the ARF gene *GH_A06G0864* probably had an impact on the number of epidermal cell protrusions in the initial stage of cotton fiber development. As mentioned in the earlier studies, AUX-IAA target genes involved in the process related to the cell wall, and the appropriate auxin level in developing the ovule or fiber, might enhance the yield and quality of cotton fibers (Chen and Guan, 2011; Nigam and Sawant, 2013). In our study, *GhIAA16* was annotated as an auxin-responsive protein IAA1-like in the Gh-Cluster7-Brown module (20 DPA), implying that the auxin-signaling pathway is involved in the late fiber elongation and SCW morphogenesis. However, the regulation network and molecular mechanism of auxin in late fiber development are not yet well-defined. Ethylene affects lots of aspects in cell differentiation, elongation, and development in different tissues of plants, including root hairs and trichomes growth in cotton fiber development (Qin and Zhu, 2011). The recent research progress associated with ethylene biosynthesis and signaling pathway proposed a possible regulatory network for cotton fiber development with ethylene as the hub, which contained *GhMAT/SAM*, *GhACS*, *GhACO*, *GhRTE, GhEBF*, and *GhEIN3/EIL* genes (Yu et al., 2021). Worthy of mention, we discovered *Gh_AACS2* (*GH_D12G0732*) which was related to ethylene synthesis in our network of Gh-Cluster7-Brown, which may promote some biochemical processes during fiber development. Yet, the regulation of cotton fiber development requires the coordination of multiple plant hormones (Shi et al., 2006), and the underlying regulatory mechanism of ethylene on the auxin pathway is largely mysterious.

### Transcription Factors With Certain Expression Patterns May Regulate the Fiber Development

TFs play regulatory roles in growth and development, morphogenesis, secondary metabolism, and stress resistance in higher plants (Shan et al., 2020). Furthermore, the development of cotton fiber is a complex and dynamic process, involving the initiation, elongation, SCW synthesis, and maturation stages, and there are a large number of genes taking part in the transcriptional regulation at different developmental periods, especially TFs (Liu et al., 2020). A total of 3,412 expressed TFs, including all the known TF families in cotton, were identified in our study, which indicates a tanglesome regulation network in cotton fiber development (Supplementary Table 4), whereas how these factors form intricate and delicate regulatory networks has not been well-elucidated. In the six new modules extracted from the co-expression networks of *G. hirsutum* and *G. barbadense*, we paid more attention to several TF families related to cotton fiber, including bHLH, MYB, HD-ZIP, TCP, and WRKY, which were well-studied in the previous reports. As we all know, the MYB family plays an essential role in regulating the initiation and elongation of cotton fibers, such as *GhMYB109*, *GhMYB25*, and *GhMYB6* (Suo et al., 2003; Machado et al., 2009; Walford et al., 2011; Wang et al., 2019b). Our results identified four MYB genes (*GH_A13G0810*, *GH_D05G2167*, *GH_A08G1565*, *GH_A11G3587*) in the new modules of *G. hirsutum*, while three of them were from the Gh-Cluster2-Blue module (–3 DPA), which suggested that the MYB factors were involved in cotton fiber cell differentiation and initiation process (Supplementary Table 10). In regulating the growth and development of plants, one of the most classical modes of transcriptional regulation is the MYB-bHLH-WD40 protein complex, which has been shown to participate in multiple aspects of plant development (Ramsay and Glover, 2005). Not surprisingly, we also found six bHLH genes (*GH_A05G0536*, *GH_D10G0727*, *GH_A07G2631*, *GH_A05G2199*, *GH_D05G0534*, *GH_A03G1665*) in the Gh-Cluster2-Blue module, implying these genes may form a pivotal regulation in fiber development (Supplementary Table 10). TCP proteins are plant-specific TFs, and perform a variety of physiological functions in plant growth and development (Li et al., 2017b). In our study, *GH_D08G2052* was identified in the Gb-Cluster4-Yellow module (10 DPA), which might contribute to the rapid elongation of cotton fiber (Supplementary Table 11). In addition, members of HD-ZIP and WRKY have also been reported to be involved in cotton fiber development, such as *GaHOX1*, *GhHD-1*, *GhWRKY16*, and *GhWRKY53* (Guan et al., 2008; Walford et al., 2012; Wang et al., 2021; Yang et al., 2021). Five HD-ZIP genes (*GH_D12G1935*, *GH_D02G1464*, *GH_A12G1753*, *GH_A01G1229, GH_A03G0781*) and three WRKY genes (*GH_D03G0476, GH_A02G1807, GH_D07G0569*) were found in our analysis (Supplementary Tables 10, 11), and the potential biological regulations of these genes in fiber development were still not clear, which could be explored for future research.

## Conclusion

In this study, time-course (MaSigPro) and co-expression (WGCNA) analyses were used to analyze systematically and comprehensively the specific or differential expression profiles of *G. hirsutum* and *G. barbadense* during fiber development. Several genes related to cell walls and auxin were predicated as possible candidates that may lead to the fiber quality differences between *G. hirsutum* and *G. barbadense*, which need further functional validation. Overall, these findings could contribute to further exploration of the cotton fiber molecular regulation mechanism, and provide the effective data support and theoretical basis for functional validation of candidate genes subsequently, which undoubtedly is of great significance for speeding up the cotton breeding and genomics research.

## Data Availability Statement

According to national legislation/guidelines, specifically the Administrative Regulations of China on Human Genetic Resources (http://www.gov.cn/zhengce/content/2019-06/10/content_5398829.htm, http://english.www.gov.cn/policies/latest_releases/2019/06/10/content_281476708945462.htm), no additional raw data is available at this time. Data of this project can be accessed after an approval application to the China National Genebank (CNGB, https://db.cngb.org/cnsa/). Please refer to https://db.cngb.org/, or email: CNGBdb@cngb.org for detailed application guidance. The accession code CNP0002656 should be included in the application.

## Author Contributions

TZ conceptualized the project. JZ performed the bioinformatics analysis. JZ, HM, and RC processed all the samples and performed the qRT-PCR. JZ, HM, HL, YH, and TZ prepared the manuscript. All authors read and approved the final manuscript.

## Conflict of Interest

The authors declare that the research was conducted in the absence of any commercial or financial relationships that could be construed as a potential conflict of interest.

## Publisher’s Note

All claims expressed in this article are solely those of the authors and do not necessarily represent those of their affiliated organizations, or those of the publisher, the editors and the reviewers. Any product that may be evaluated in this article, or claim that may be made by its manufacturer, is not guaranteed or endorsed by the publisher.
