# Supplementary material for: Transcriptome Time-Course Analysis in the Whole Period of Cotton Fiber Development
Source: Front Plant Sci. 2022 Apr 5;13:864529. doi: 10.3389/fpls.2022.864529 (PMC9022538; doi:10.3389/fpls.2022.864529)
Supplement: Supplementary file 14 [file Data_Sheet_1.docx]

**Transcriptome time-course analysis in the whole period of cotton fiber development**

Juncheng Zhang, Huan Mei, Hejun Lu, Rui Chen, Yan Hu, Tianzhen Zhang^*^

Zhejiang Provincial Key Laboratory of Crop Genetic Resources, Institute of Crop Science, Plant Precision Breeding Academy, College of Agriculture and Biotechnology, Zhejiang University, Hangzhou, China

*Correspondence and requests for materials should be addressed to Tianzhen Zhang ([cotton@zju.edu.cn)](mailto:cotton@zju.edu.cn)).

**Supporting Figure S1- S5**

Figure S1 Principal component analysis of all samples.

Figure S2 Principal component analysis of three cultivars respectively, and pearson correlation coefficient analysis between *G. hirsutum* and *G. barbadense*..

Figure S3 GO enrichment analysis results of six new modules.

Figure S4 Gene co-expression correlation networks of six new modules.

Figure S5 RNA-seq expression heatmap of candidate genes in At or Dt subgenome.


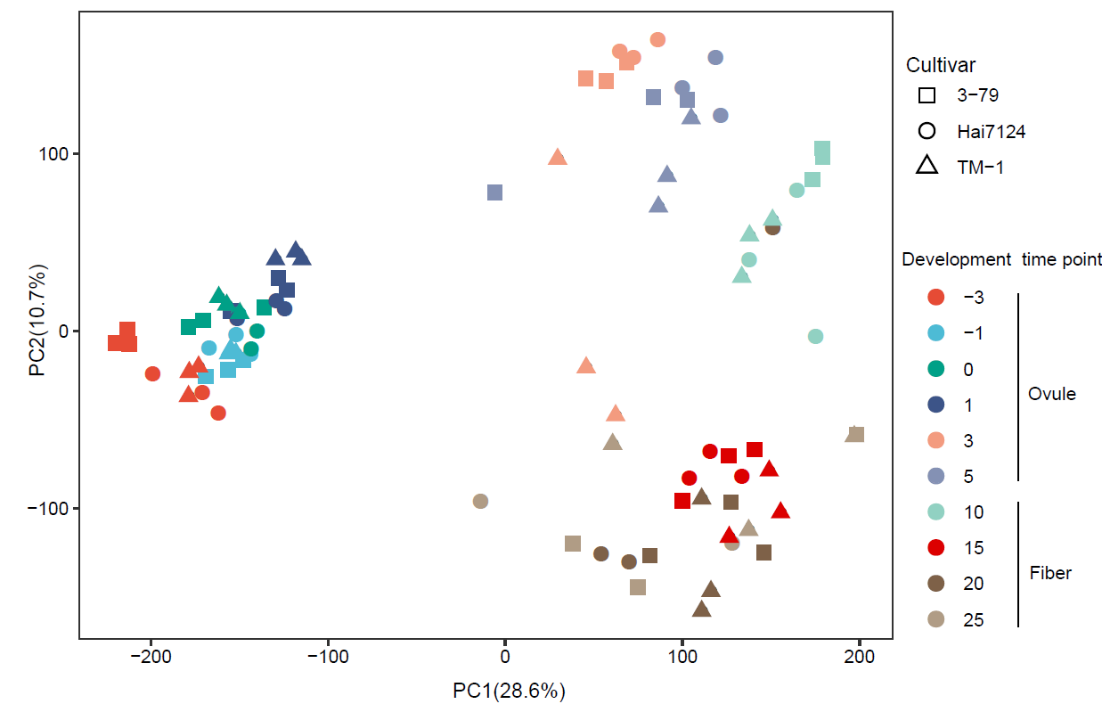


Figure S1 Principal component analysis of all samples. Different colors represent samples at different development time points, and different shapes represent cultivars.


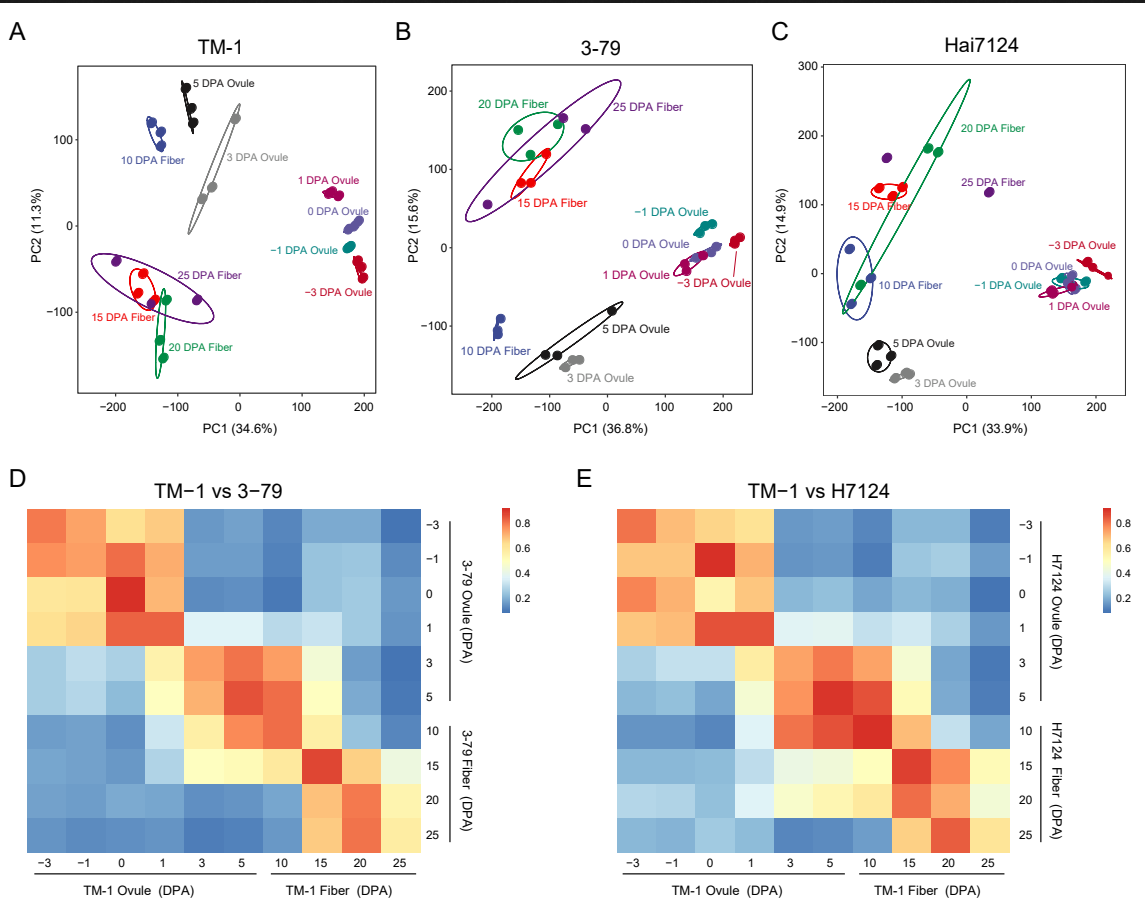


Figure S2 (A-C) Principal Component Analysis of *G. hirsutum* TM-1, *G. barbadense* 3-79, *G. barbadense* Hai7124. Different colors represent samples at different development time points. Ellipses reflect a 68% confidence interval around the colored group centroid. (D) Pearson Correlation Coefficient of 3-79, TM-1. (E) Correlation Coefficient of Hai7124, TM-1**.**


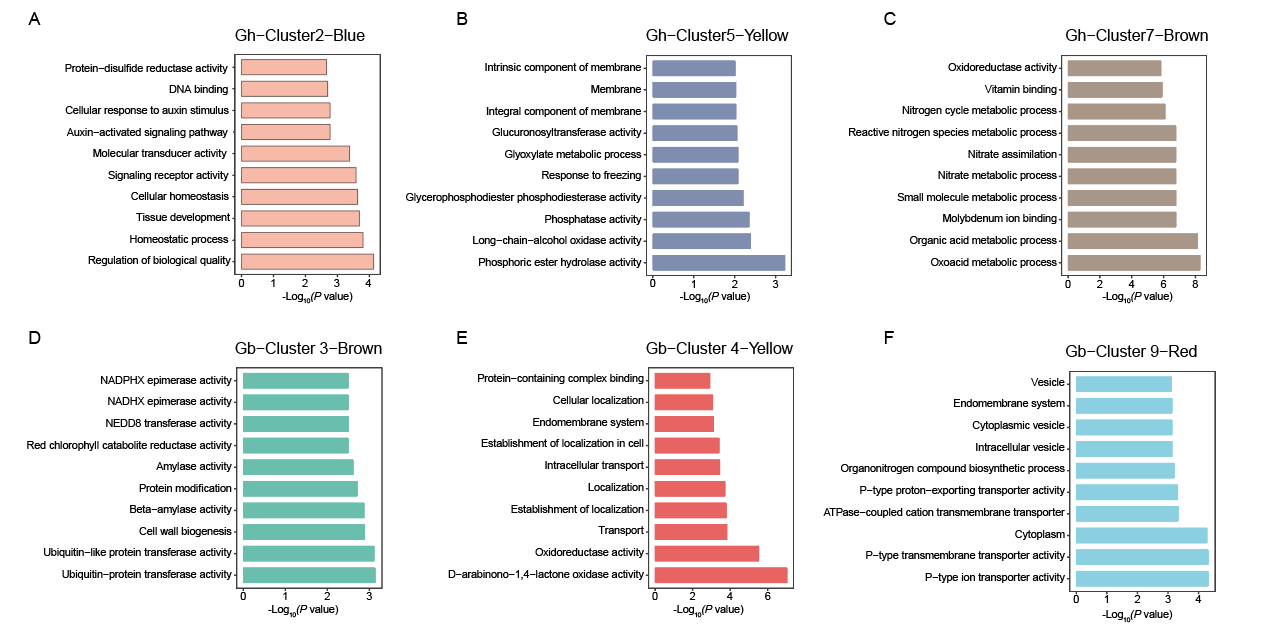


Figure S3 GO enrichment of Gh-Cluster2-Blue (A), Gh-Cluster5-Yellow (B), Gh-Cluster7-Brown (C), Gb-Cluster3-Brown (D), Gb-Cluster4-Yellow (E), Gb-Cluster9-Red (F). The different colors represent different new modules. The X‐axis represents -log_10_(*P*‐value) and enriched GO terms are indicated on the Y-axis.


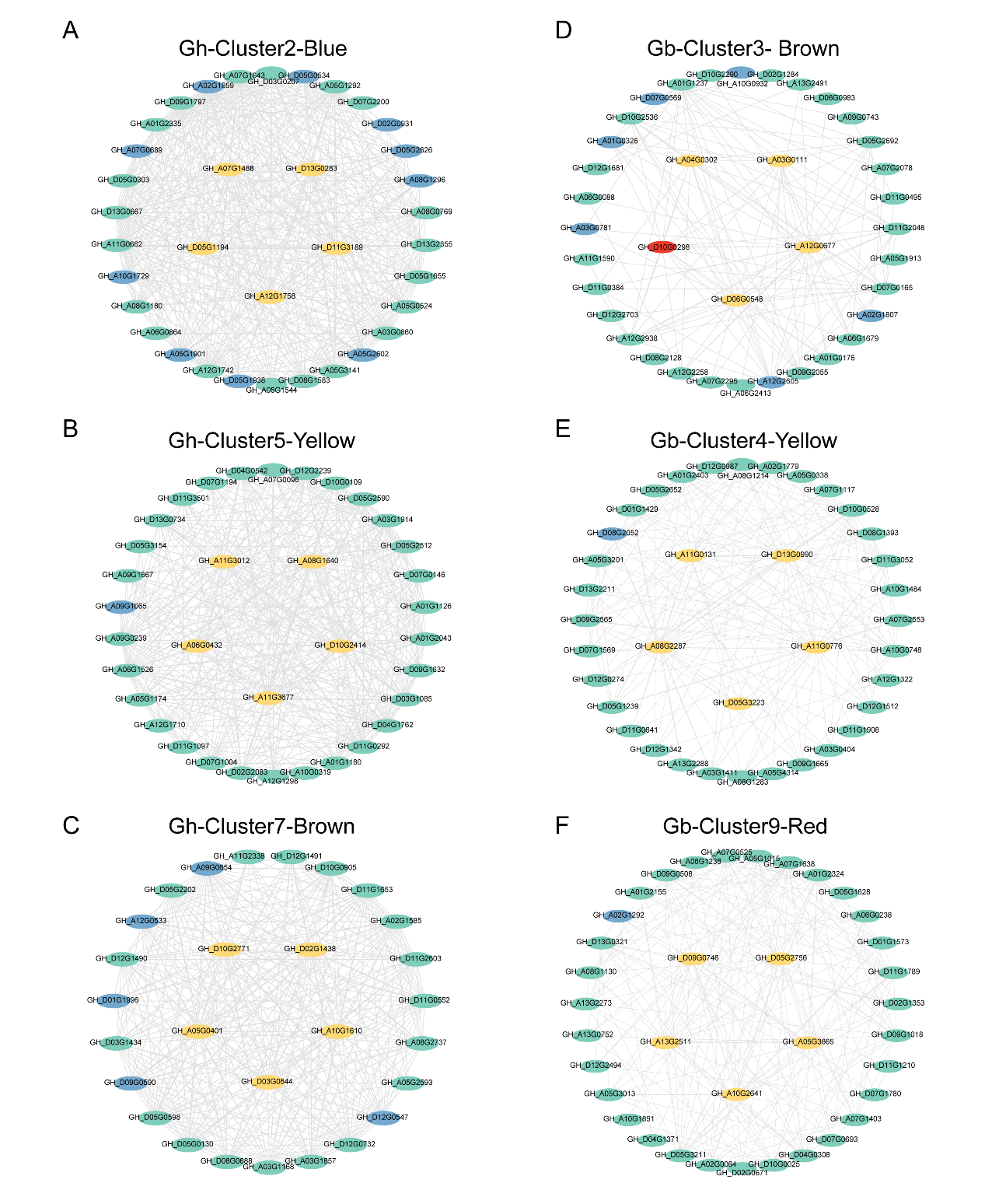


Figure S4 Gene co-expression correlation networks for Gh-Cluster2-Blue (A), Gh-Cluster5-Yellow (B), Gh-Cluster7-Brown (C), Gb-Cluster3-Brown (D), Gb-Cluster4-Yellow (E), Gb-Cluster9-Red (F). Hub genes are indicated by yellow circles; blue circles indicate transcription factors; red circles indicate both a hub gene and a transcription factor; green circles indicate associated genes.


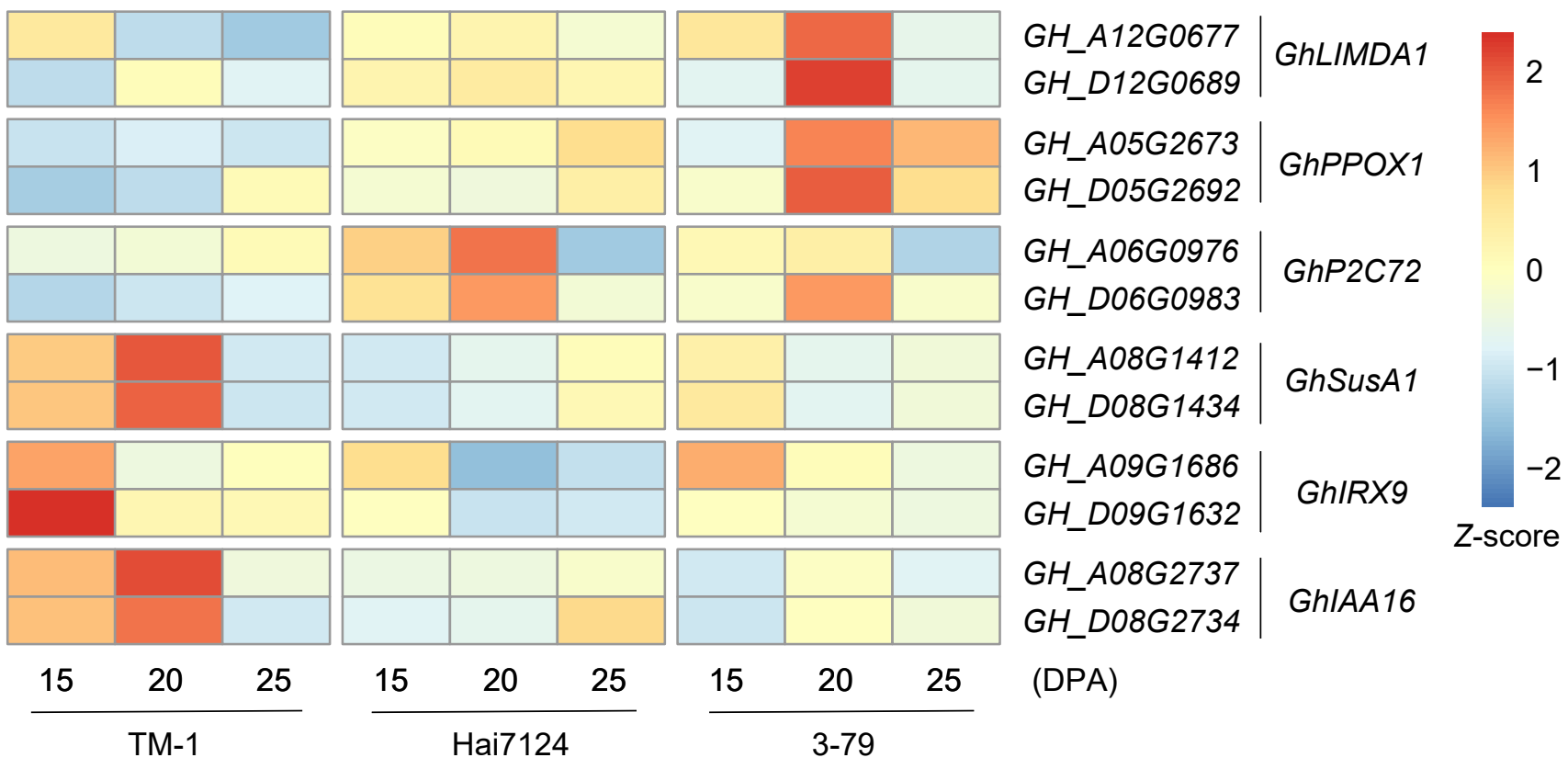


Figure S5 RNA-seq expression heatmap of candidate genes in At or Dt subgenome. The abscissa represents the three cultivars at 15, 20, and 25 DPA. The legend indicates that the expression level FPKM is normalized (*Z*-score) by row.
